# P48 Lupus with a neurological twist: from skin rash to sudden confusion and a diagnosis of posterior reversible encephalopathy syndrome

**DOI:** 10.1093/rap/rkae117.079

**Published:** 2024-11-05

**Authors:** Yuva Ravindran, Aveen Mahmood, Nicola Erb

**Affiliations:** The Dudley Group NHS foundation trust, Dudley, United Kingdom; The Dudley Group NHS foundation trust, Dudley, United Kingdom; The Dudley Group NHS foundation trust, Dudley, United Kingdom

## Abstract

**Introduction:**

Neuropsychiatric lupus (NPSLE) is a severe complication of systemic lupus erythematosus (SLE), comprising a spectrum of neurological and psychiatric manifestations. NPSLE poses diagnostic challenges due to lack of specific biomarkers to aid diagnostic confirmation and overlapping symptoms with many primary neuropsychiatric conditions. Posterior reversible encephalopathy syndrome (PRES) is a syndrome where patients present acutely or sub acutely with altered consciousness, visual disturbances, seizures, headache and typical MRI findings and can be triggered by a diverse range of precipitants. In this case, we report an atypical presentation of PRES in a patient with new-onset lupus alongside hydroxychloroquine/lupus induced erythema multiforme.

**Case description:**

A 78-year-old female, recently diagnosed with SLE with cutaneous manifestations and positive ANA and dsDNA antibodies, presented with three days of worsening confusion and widespread erythematous rash. Examination on admission suggested hypoactive delirium and an extensive rash. Blood investigations showed severe hyponatremia and normal inflammatory markers. She was treated with hypertonic saline, empirical antibiotic and antiviral treatment for probable meningoencephalitis. Dermatology opinion was sought for the rash and was initially suspected to be erythema multiforme induced by hydroxychloroquine (HCQ) or secondary to SLE itself. Investigations with tumour markers and CT imaging did not identify any malignancy to suggest the rash to be paraneoplastic. However, skin biopsy revealed it to be erythema multiforme, which then settled with withdrawal of hydroxychloroquine and administration of steroids. Within 48 hours, her confusion and hyponatremia resolved. Initial CT head showed no abnormality.

However, her confusion recurred with hypokalemia, right-sided gaze deviation, clinical cortical blindness and persistent severe hypertension. Lumbar puncture was not performed due to the development of thrombocytopenia. MRI brain demonstrated extensive T2/FLAIR signal changes in the cerebral hemispheres and bilateral asymmetrical cerebral white matter, with sulcal effacement and extensive supra- and infratentorial patchy hemorrhagic changes and widespread SWI changes. Differential diagnosis included vasculitic pathology, cerebral-amyloid-angiopathy associated inflammation, thrombotic thrombocytopenic purpura(TTP) and PRES. Blood film revealed no microangiopathic hemolysis. CT angiogram showed multifocal high attenuation in right temporal, occipital and parietal lobes. Further review with the neuro-radiology team suggested the images were consistent with PRES with haemorrhage. She was treated supportively with antihypertensives, intravenous and then oral corticosteroids, and later started on mycophenolate mofetil. Repeat MR brain showed some resolution of changes. She continued to have fluctuating confusion over the next two months, and ultimately made full recovery with no further neurological concerns.

**Discussion:**

Neuropsychiatric SLE includes clinical manifestations that range from minimal cognitive decline to seizures and psychosis. The American college of Rheumatology identified 19 NPSLE syndromes including 12 central nervous system (CNS) and 7 peripheral nervous system ones. Posterior reversible encephalopathy syndrome (PRES) was not defined as an NPSLE but is being increasingly identified as a condition linked to autoimmune diseases including SLE. PRES is a clinico-radiological diagnosis, where patients present with a rapid onset combination of global encephalopathy, seizures, headache, or visual symptoms. As the clinical presentation of PRES is highly non-specific, the diagnosis relies upon typical MRI findings of vasogenic oedema in posterior cerebral cortex, best seen with FLAIR sequencing. PRES has many underlying causes, the commonest being poorly controlled hypertension particularly in the setting of pre-eclampsia. Other frequent causes include metabolic and electrolyte disturbances, medication including immunosuppressants and sepsis. Autoimmune diseases linked to PRES include SLE, Rheumatoid arthritis and Crohn’s disease. However, less than 0.8% of PRES have been associated with autoimmune diseases.

The acute management of PRES is supportive, involving correction of hypertension and removal of suspected precipitating factors. Prognosis, like in our patient, is excellent with timely and prompt management of blood pressure and treatment of any additional precipitating factors.

This case also highlighted the diagnostic uncertainty of cutaneous SLE. The patient’s biopsy findings were suggestive of erythema multiforme, secondary to SLE or medications. Hydroxychloroquine could trigger erythema multiforme and management typically involves removal of the offending drug. However, in our case, due to the simultaneous discontinuation of the offending drug and the treatment of underlying lupus, the diagnostic uncertainty persists.

**Key learning points:**

• Systemic lupus erythematosus has been considered to be a disease of young women traditionally, however in 10-20% patients, the diagnosis can be made in the fifth decade of life or later. Clinical manifestations in older population can be different from that of the typical cohort and diagnosis of neuropsychiatric lupus is especially challenging in older patients because of increased incidence of neurological co-morbidities and a higher likelihood of underlying CNS infections. Steroid psychosis can be a confounding factor for NPSLE. NPSLE can also be different from other complications of lupus as it may develop without serological changes and frequently manifests with active SLE.

• PRES is not a common manifestation of NPSLE and is usually diagnosed easily when there is an identifiable known trigger such as eclampsia or hypertension. However, connective tissue disorders should be considered as differential diagnoses. PRES can be the initial manifestation of neurolupus, although not commonly seen. The exact pathophysiological mechanism of PRES remains uncertain. The imaging changes on MRI in PRES often mimic vasculitic changes and specialist neuroradiology review of imaging may often be needed. In this patient, haemorrhaging was extensive in the supratentorial distribution but commonly PRES is associated with intraparenchymal bleed. Complete reversibility is regarded as a defining feature of PRES, however, lesions can progress to irreversible damage and leukomalacia necessitating the need for regular follow up and imaging. The ideal timing of repeated brain imaging to document recovery remains unclear.

• Cutaneous lupus is another complex entity and presentations with erythema multiforme are quite uncommon. With a potential trigger such as hydroxychloroquine, the diagnosis remains a mystery in our case.

• Currently NPSLE is a diagnosis of exclusion and expert opinion. High suspicion and thorough assessments are needed and although PRES can be life threatening, timely identification and management have proven to be extremely effective.

